# Titanium dioxide nanoparticles in oncology: synthesis, application, and challenges

**DOI:** 10.1007/s10565-026-10190-3

**Published:** 2026-04-17

**Authors:** Parth Agarwal, Rachana Raman, Rushil Dalal, Harshil Jain, Ajaatshatru Sisodia, Prasoon Agarwal, Praveen Kumar

**Affiliations:** 1 Manipal Institute of Technology, Manipal Academy of Higher Education, Manipal, India; 2 SciLifeLab, Division of Occupational and Environmental Medicine, Department of Laboratory Medicine, National Bioinformatics Infrastructure Sweden (NBIS), Lund University, 22362 Lund, Sweden

**Keywords:** Nanomedicine, Targeted Drug Delivery, Oncotherapeutics, Tumor Microenvironment, Immunotherapy, Green Synthesis

## Abstract

Nanoparticles (NPs) are defined as particles that have a diameter less than 100 nm. Titanium dioxide (TiO_2_) NPs (TiO_2_ NPs) have gained significant attention in recent years as a possible therapeutic agent due to their unique physiochemical properties. They include tunability of the NPs, effective dopant compatibility, biocompatibility and catalytic properties. The shape and size of the NPs also play an important role in TiO_2_ NPs for its efficiency in drug encapsulation, circulation time, efficacy and aggregation. The most effective size for TiO_2_ NPs ranges from 20-150 nm, with the preferred shape being spherical. TiO_2_ NPs, although largely ineffective as the sole therapeutic agent, have shown the ability to reduce tumorigenesis and augment the efficacy of cancer therapy in a range of cancers and treatments, such as photodynamic therapy (PDT), sonodynamic therapy (SDT), photothermal therapy (PTT), immunotherapy, and chemotherapy. However, TiO_2_ NPs have been associated with cytotoxic and genotoxic effects in several settings, which are discussed in this review. Regulatory barriers hinder its applicability, and are directly related to long-term toxicity due to prolonged exposure. These obstacles have hindered translation of these NP-based technologies in clinical settings. This highlights the need to conduct further research to address possible concerns regarding toxicity and safety even after long-term exposure. In this review, we discuss the mechanism of TiO_2_ NPs as a DDA, its applications in cancer therapy, potential risks, as well as strategies to minimize its associated risks.

**Graphical Abstract:**

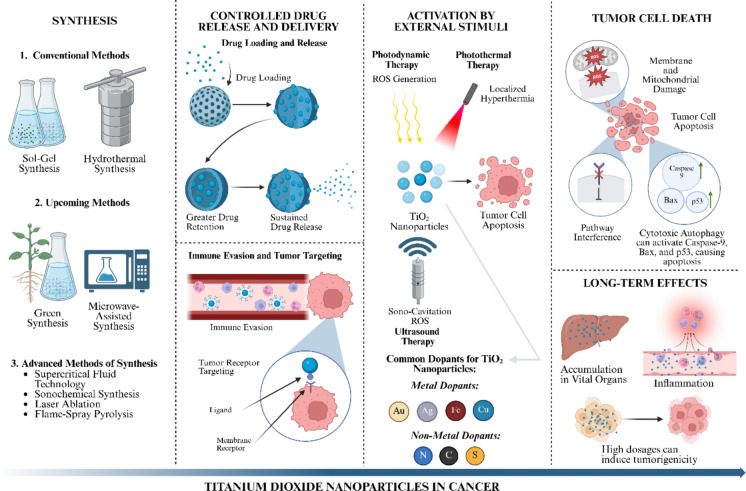

## Introduction

Over the years, various strategies for drug delivery strategies have been developed to target tumor cells in cancer therapy. However, many of these agents lack precise control and can result in side effects in patients. (Wang et al. 2015). A viable option to overcome these limitations is the use of nanotechnology in cancer studies. Other approaches include targeted antibody–drug conjugates, biomimetic carrier-based therapies, and immune-based therapies (Long et al 2025; Valcourt et al. 2018, 2018). A nanoparticle can be defined as a nanosized particle that has dimensions smaller than 100 nm, and as the particles approach nanoscale, they display unique properties that differ significantly from their bulk forms (Bogdan et al. 2017). One such property is the recognition of steric stabilization, which creates a repulsive force between particles, thereby hindering particle aggregation. This makes them ideal for use in drug delivery systems (DDS) (Sultana, et al. 2020). Furthermore, the morphology of NPs plays a critical role in determining their therapeutic efficiency; For example, spherical NPs remain the most widely used form due to their narrow size distribution, high surface area, adjustable surface chemistry, and suitability for drug encapsulation and delivery (Hasanzadeh Kafshgari and Goldmann 2020). Notably, spherical NPs within the size ranges of 20–150 nm exhibit lower agglomeration and greater biodistribution across major organs such as liver, lung, kidneys, and spleen, making them suitable for therapeutic purposes (Bhullar et al. 2022a). Additionally, their shape supports prolonged circulation and enables them to effectively deliver drugs as a result of the enhanced permeability and retention (EPR) effect, making them suitable for cancer therapy (Yao et al 2020). Amongst the various NPs investigated for such applications, TiO_2_ NPs possess several outstanding characteristics such as biocompatibility, corrosion resistance, high photo-activity, and chemical stability. They are also highly effective as anti-microbial, anti-bacterial, and anti-cancer agents (Shiva Samhitha et al. 2022).

TiO_2_ has three different crystal structures: rutile, brookite, and anatase, with the anatase form being the most commonly used due to its high photocatalytic activity (Ansari and Daneshjou 2024). Promising applications of TiO_2_ NPs in cancer therapy include their use in PDT, PTT, SDT, as well as a DDA for anti-cancer drugs, such as paclitaxel, temozolomide, doxorubicin, camptothecin, and daunorubicin (Behnam et al. 2018). TiO_2_ NPs were found to induce changes in gene expression, metabolic processes, inflammatory responses, and homeostasis, while also causing DNA damage in HepG2 tumor cells (Zarzzeka, et al. 2023). The activation of TiO_2_ NPs by light or ultrasound waves allows for the generation of reactive oxygen species (ROS) or heat, which, in turn, induces cell damage and thus causes the death of tumor cells. Additionally, TiO_2_ NPs have been used for treatment in several cancers, such as breast cancer, melanoma, and lung cancer, where it has shown promising results (Zarzzeka, et al. 2023). Beyond therapy, TiO_2_ NPs have also shown potential in diagnostic imaging applications, and when combined with therapeutic applications, they can function as multifunctional platforms that enable simultaneous diagnosis and treatment of cancer (2021).

Growing evidence also highlights potential toxic side-effects of TiO_2_ NPs in humans. In vivo and in vitro studies have shown that cytotoxicity and genotoxicity are induced by TiO_2_ NPs in sperm cells, plasma cells, epithelial cells, endothelial cells (Santonastaso et al. 2020). Moreover, due to their small size, TiO_2_ NPs have also been found to be capable of crossing the blood brain barrier, subsequently inducing ROS generation, protein oxidation, and impairment of antioxidant mechanisms (Baranowska-Wójcik et al. 2020). However, most of the deleterious effects of TiO_2_ NPs, such as DNA damage, alteration of biochemical parameters, and tissue damage are dependent on particle size and dosage (Jovanović 2015). Furthermore, prolonged exposure to TiO_2_ NPs has been shown to trigger inflammatory responses in healthy tissues, raising suspicion about their systemic toxicity (Medina-Reyes et al. 2015). Various strategies have been developed to mitigate these negative side-effects. Polymer coating of TiO_2_ NPs is considered to be the most effective method in regards to keeping toxicity levels low, and since polyethylene glycol (PEG) is an FDA-approved, affordable, well-characterized polymer, it is usually preferred for nanoparticle coating (Jokerst et al. 2011). Other methods that have been explored to lower TiO_2_ NP toxicity include surface modification, target functionalization, and controlling dosage levels (Thiruppathi et al. 2017; Hajareh Haghighi, et al. 2023; Waani et al. 2021). Given these properties, and its unique therapeutic versatility, this review provides a comprehensive and up-to-date overview of the synthesis, functionalization, therapeutic applications, and long-term effects of TiO_2_ NPs in the context of oncology.

## Synthesis of TiO_2_ NPs

The performance of TiO_2_ NPs largely depends on the fabrication of the NPs and their resulting morphology. Their size, shape and surface characteristics can be engineered in a highly precise manner, resulting in the NPs having dynamic physicochemical properties. However, as these NPs are non-degradable, it is essential to minimize the risk of agglomeration and assure accelerated clearance when used as drug delivery agents (DDAs) (Hasanzadeh Kafshgari and Goldmann 2020). The properties of TiO_2_ NPs depend on the method of synthesis used. Therefore, different synthesis methods are used to tailor TiO_2_ NPs to specific applications. NP synthesis can occur in a gaseous, liquid or solid medium, with the penetration rate of the reactant in a gas or liquid phase being faster than in the solid phase (Hsu et al. 2024). Commonly used media in different phases include inert gases for vapor-phase synthesis, aqueous or organic solvents for liquid phase methods, and solid matrices for solid-state synthesis(Kumari et al. 2023). A commonly used precursor reactant is titanium isopropoxide (TTIP), which is preferred due to its high reactivity and suitability for hydrolysis (Vinukonda et al. 2025). These properties enable the controlled nucleation and growth of NPs under mild conditions of temperature and pressure. It also allows for control over particle properties such as size and morphology (Chang et al. 2023). In addition to the chosen synthesis method, parameters such as pH, temperature, precursor concentration, and reaction time play an important role in determining the properties of NPs. Higher precursor concentrations generally promote rapid particle growth and aggregation, resulting in larger NPs. On the other hand, lower concentrations favor controlled nucleation and formation of smaller, more uniform particles (Haydar et al. 2025; Kazemi et al. 2023). The sol–gel and hydrothermal methods are the most commonly used methods of synthesis, while green chemistry synthesis has been gaining traction in recent years (Ziental et al. 2020). These methods of synthesis will be discussed in detail in the following sections. A comparative summary of these synthesis methods, including their synthesis conditions, scalability, reproducibility, advantages, and limitations is presented in Table 1.

**Table 1:** Comparative overview of TiO_2_ NPs synthesis methods

Method of Synthesis	Synthesis Conditions	Scalability and Reproducibility	Advantages	Limitations	References
Sol–Gel Synthesis	Variable temperature; key conditions include humidity, stirring rate, sequence of chemical addition, and aging time	Scalability is limited due to long gelation and drying times Additionally, reproducibility is limited due to high sensitivity towards reaction conditions such as pH, temperature, and precursor concentration	• Low processing temperature • Ability to form complex structures • Uniformity of the NP • High purity • Complete control over particle size and morphology	• Long processing time required for gelation and drying • Environmental and safety concerns due to use of organic solvents • Shrinkage and cracking of the gel, leading to defects in NP structure	Vinukonda et al. 2025; Chang et al. 2023; Zuo et al. 2024)
Hydrothermal Synthesis	High temperature, high pressure, takes place in a stainless-steel vessel. It is heated in an autoclave lined with Teflon. Synthesis conditions such as pH and precursor concentration control formation chemistry	Scalability is limited due to need for specialized autoclaves. Reproducibility is limited by sensitivity to solvent type, precursor concentration, and reaction time	• High purity • Freedom to adjust reaction conditions • High crystallinity • Environmentally friendly • Synthesis of materials that are unstable at higher temperatures	• Process is difficult to control	Vinukonda et al. 2025; Zuo et al. 2024; Gupta et al. 2021; Gan et al. 2020; Tavakoli et al. 2007)
Green Synthesis	Room temperature, key conditions include type and concentration of plant extract, precursor concentration, pH, stirring rate, and calcination temperature	Small-scale production has been found to be cost-effective and feasible. However, large-scale synthesis is in its early stages and limited by slower reaction kinetics, and yield challenges Reproducibility is an additional challenge due to discrepancies in plant species, cultivation, and plant extraction	• Use of renewable resources • Sustainable process • Easy handling • Non-toxic compounds • Biocompatibility	• Contamination risk • Variation in reproducibility due to use of different phytochemicals • Complexity of reaction conditions	Eddy, et al. 2024; Bhullar et al. 2021; Saxena, et al. 2025; Shakeel et al. 2025)
Microorganism-Mediated Synthesis	Variable temperature (organism-dependent), key conditions include type of microorganism being used, nature of precursor and its concentration, pH, and incubation time	Variation in microbial strains, growth conditions, metabolic activity between batches affect reproducibility Large-scale synthesis is affected due to difficulties related to oxygen transfer, nutrient availability, and pH regulation	• Biocompatibility • Environmentally friendly nature • Non-toxicity • Resistance to aggregation • Increased Stability	• Tendency to polydisperse • Difficult to purify	Ghareeb et al. 2024; Rathore et al 2023; Carmona et al. 2023; Campaña et al. 2023; Akdaşçi et al. 2025)
Microwave-Assisted Synthesis	Varied electromagnetic frequency, heating occurs via ionic conduction and dipolar polarization. Key conditions include microwave power, exposure time, and reaction time	Can be reproduced easily at laboratory scale with dedicated microwave instruments Large-scale production is limited by the low penetration ability of microwave irradiation, increased heat loss, and absorption changes	• Faster reaction times • Energy efficient • Higher yields compared to traditional methods	• Intense heat from the process may disrupt pathway and product distribution • Non-uniform heating may lead to incomplete product formation	Arun et al. 2023; Kubiak and Cegłowski 2024; Diaz-Ortiz, et al. 2011)
Supercritical Fluid Technology	Supercritical conditions required, key conditions include pressure, temperature, and choice of supercritical fluid. The precursor is injected into a high-pressure reactor	Enhanced reproducibility due to precise control over temperature and pressure More sustainable scale-up compared to other techniques due to greater resource efficiency, making it economically viable	• Low cost and simplicity • Carbon dioxide and water can be used as a supercritical fluid instead of organic solvents • Faster synthesis of NPs compared to conventional methods	• Requires high pressure and critical temperature for NP synthesis	Jamkhande et al. 2019; Sankula et al. 2014; Philippot et al. 2014; Andrés et al. 2026)
Sonochemical Synthesis	Ultrasonic irradiation at various frequencies, key conditions include duration of sonication, precursor concentration, and temperature of the reaction medium	Strong reproducibility as particle properties as maintained despite different reactor volumes Scalability is also viable due to constant radical generation across different volumes, although larger reactions may exhibit greater cavitation fields	• Enhanced photocatalytic capabilities due to greater surface area • Eco-friendly • Cost-effective	• NPs cannot be consistently reproduced • Rate of sonochemical reaction is dependent on ultrasonic frequency	Najafidoust et al. 2026; Matyszczak et al. 2024; Bang and Suslick 2010; Hansen et al. 2024)
Laser Ablation	Pulsed laser irradiation of a solid target submerged in a liquid medium. Key conditions include ablation time, choice of liquid medium, laser pulse duration	Reproducibility is high due to high controllability of laser, target and liquid parameters, which ensures consistent NP properties Scalability is limited due to low production	• Simple and effective technique • Allows for NP formation without surfactants • Precise control over NP properties	• Prolonged ablation forms a colloidal solution that blocks the laser path, reducing ablation rates	Jamkhande et al. 2019; Simakin et al. 2004; Ghorbani 2014; Hansen et al. 2024)
Flame-Spray Pyrolysis	High temperature combustion, key conditions are the diameter of the precursor droplet, residence time of particles in the flame, and configuration of the flame spray pyrolysis system	Highly reproducible due to flame-driven particle formation, which allows for control over NP properties, allowing them to stay consistent Scalability is favorable due to continuous, high-throughput production, fast reaction times, and no toxic byproducts	• Short manufacturing time • High reproducibility • Doesn’t use liquid byproducts that are difficult to dispose	• Low NP purity • Poor control over NP properties • Parameters for pyrolysis can be quite complex	Alhaleeb and Machin 2020; Teoh et al. 2010; Li et al. 2026; Xin et al. 2025)

### Sol–gel method

The sol–gel method involves reactions focused on hydrolysis and polycondensation, during which Ti–O-Ti bridges are formed. For the specific synthesis of TiO_2_ NPs, titanium alkoxide precursors, such as titanium butoxide or titanium (IV) isopropoxide, are added to the reaction system alongside acid/water and alcohol. After the mixture is stirred for several hours, the formation of dense, cross-linked, three-dimensional structures take place which results in the formation of a TiO_2_ gel (Zuo et al. 2024). The reaction mechanism is illustrated below (R = alkoxyl group, OR = alkoxide group, x = number of groups):

Hydrolysis Reaction:

1 $$Ti (OR)x + H2O \to Ti (OH)(OR)x-1 + ROH$$

2 $$Ti (OR)x-1 + H2O \to Ti (OH)(OR)x-2 + ROH$$

3 $$This continues until: Ti (OH)x$$

Polycondensation Reaction:

4 $$-Ti-OH+HO-Ti- \to -Ti-O-Ti- + H2O$$

5 $$-Ti-OR- + HO-Ti- \to Ti-O-Ti + ROH$$

After the sol–gel process is complete, the TiO_2_ gel is must be aged, the time for which can range from several hours to several days, depending on the required properties of the NPs. Lastly, the gel is dried to remove any remaining solvents or alcohol, via methods such as evaporation, freeze drying, and supercritical drying to obtain a solid form of the NP (Chang et al. 2023). The sol–gel method for synthesis is widely used due to its simplicity, speed, and low cost. Additional advantages include low processing temperature, ability to form complex structures, the uniformity of the NP thus formed, high purity, and the ability to have complete control over the particle size and morphology (Vinukonda et al. 2025; Zuo et al. 2024). The sol–gel method can also be used to effectively synthesize inorganic nanoplatforms with integrated drug release mechanisms, highlighting the adaptable nature of this method in integrating newer modifications (Sanattalab et al. 2023).

### Hydrothermal synthesis

Hydrothermal synthesis refers to the preparation of TiO_2_ NPs through the utilization of crystal growth patterns, dissolved titanium salts, organic compounds containing titanium or titanium oxide compounds, and occasionally, additives such as alkalis. This process is carried out in a closed system under high temperature and pressure condition. There are three main steps in hydrothermal synthesis: growth of the crystal, transformation of the crystal, and establishment of physical equilibrium (Zuo et al. 2024). The synthesis takes place in an autoclave lined with Teflon, where the crystal is placed in a stainless-steel vessel and heated to high temperatures using a muffle furnace (Gupta et al. 2021). By changing the ratio of additives or titanium sources added, along with the reaction time, temperature, and nucleation rate, the properties of the NPs can be customized for specific requirements (Hsu et al. 2024). Advantages of the hydrothermal method include cost effectiveness, the ability to form high purity NPs, and the customizability of the set reactions to meet specific requirements (Gupta et al. 2021). Additional advantages include the synthesis of materials that are unstable at higher temperatures, high crystallinity, and the environmentally friendly nature of the synthesis process (Gan et al. 2020). However, the waste generated as a byproduct during this process is potentially toxic to the environment, which can offset the benefits.

Despite the popularity of the sol–gel and hydrothermal methods for NP synthesis, they both require high operating temperatures and pressures while also being potentially toxic to the environment, if the techniques are not managed properly (Zuo et al. 2024).

### Green synthesis

Green synthesis is an environmentally friendly and cheaper alternative for NP synthesis and has thus become an attractive method for researchers in recent years(Selvaraj et al. 2022). Plant extraction is also a natural eco-friendly alternative to conventional chemical reagents as it involves the isolation (and potential repurposing) of secondary metabolites, such as polyphenol. Various methods exist for polyphenol extraction and are largely dependent on the type of polyphenol being extracted. Examples of extraction methods include ultrasonic-assisted extraction (UAE), microwave-assisted extraction (MAE), supercritical fluid extraction (SFE), and pressure-assisted liquid extraction (PALE) (Eddy, et al. 2024). The synthesis of the TiO2 NPs involves two steps: preparation of the plant extract and synthesis of the NP. A titanium precursor solution with the desired solvent is required for this process. Common solvents used here are ethanol or water, while titanium (IV) isopropoxide is a commonly used precursor (Bhullar et al. 2021). The plant extract is added dropwise while simultaneously stirring the solution at room temperature. Following this, the NPs are filtered prior to the calcination that takes place at temperatures ranging from 400–800 °C. Calcination is essential to remove organic groups (Eddy, et al. 2024). Building on these procedures, emerging methods such as microorganism-mediated and microwave-assisted synthesis offer greater control and efficiency. Other extraction techniques use cheap precursors, such as *Solanum Tuberosum* extract-derived TiO2 nanoparticles, cementing green synthesis as a promising, low-cost and accessible synthesis method (Girigoswami et al. 2024).

### Emerging and advanced methods of synthesis

#### Microorganism-mediated synthesis

Microorganisms, such as bacteria, fungi, and algae produce a variety of pigments, metabolites, and enzymes that can be used for NP synthesis. The advantages of this method include biocompatibility, its eco-friendly nature, and non-toxicity (Ghareeb et al. 2024; Rathore et al 2023). This synthesis method involves two major steps: microbial culture preparation and NP synthesis. First, appropriate microbial strains are isolated and cultured under suitable growth conditions. Following this, a titanium precursor is added to the microbial culture. Microorganisms generally synthesize NPs through extracellular and intracellular modes. During intracellular synthesis, Ti3 + ions are taken up by microbial cells and reduced enzymatically within the cell wall, thereby leading to NP formation. In contrast, the extracellular method involves the secretion of enzymes or biomolecules into the surrounding medium, where Ti3 + ions are reduced and then form NPs outside the cell (Rathore et al 2023). Bacteria are the most efficient option due to easy genetic transmission, and economical costs. Common bacteria used here include *Lactobacillus* sp., *Bacillus Subtilis*, and *Aeromonas Hydrophilla* (Singh Jassal et al. 2022). Fungi, however, are also widely used due to their ability to secrete a large number of enzymes, the large surface area, and relatively simple downstream processing. In comparison to bacteria, fungi have received increasing attention as it is easier to extract and purify the required components from fungal bodies (Aziz et al. 2019; Irshad et al. 2021). Overall, microorganism-mediated synthesis offers a scalable, eco-friendly, and biologically sustainable platform for TiO_2_ NP synthesis.

#### Microwave-assisted synthesis

Microwave-assisted synthesis is an emerging technique that uses electromagnetic waves with frequencies in the ranges of 0.3–3000 GHz and wavelengths of 0.001–1.001 nm to heat reaction mixtures and facilitate TiO_2_ NP synthesis. Heating occurs through two mechanisms: ionic conduction and dipolar polarization. Here, polar molecules generate heat via rotation, friction, and collision as they try to align with the rapidly alternating electric field, simultaneously conducting ions generate heat due to collisions and directional movement (Arun et al. 2023). The method has gained attention due to faster reaction times, energy efficiency, and higher yields when compared to traditional methods. However, this method also has its disadvantages. The intense heat generated by the process can lead to disruption in pathway function and product distribution. Additionally, non-uniform heating can lead to incomplete product formation (Kubiak and Cegłowski 2024). Recent techniques have attempted to fix these shortcomings by shifting the attention towards improving control over crystallization dynamics. A 2024 study notably introduced an in-situ microwave-assisted crystallization approach, which allows for anatase TiO_2_ NP formation at temperatures lower than 100 °C. This approach showed greater photocatalytic and photovoltaic activity. This demonstrated that controlling microwave radiation could possibly reduce thermal disruption, while still having the same advantages (Kubiak et al. 2024).

#### Supercritical fluid technology

Supercritical fluids (SCFs) are widely employed in chemical and material sciences for the synthesis of NPs due to their tunable physiochemical properties (Jamkhande et al. 2019). Commonly used SCFs include carbon dioxide, water, nitrous oxide, ethanol, methanol, ethane, propane, ammonia, and n-hexane (Sekhon 2010). A key characteristic of SCFs is their ability to transition between gas-like and liquid-like properties through adjustment to pressure or temperature, which enabled precise control over conditions such as surface tension, diffusivity, viscosity, and density (Jamkhande et al. 2019). The ability of SCFs to rapidly expand is one of the main features in their application for NP synthesis (Ye and Wai 2003). In this method, nonaggregated TiO_2_ NPs are formed by coating them with a protective layer that attaches to their surface, quenches particle growth, and prevents aggregation. The synthesis occurs in a reactor equipped with high-pressure pumps and controlled by a backpressure regulator valve. The titanium precursor is dissolved in an appropriate solvent and injected into the reactor at supercritical conditions. The resulting TiO_2_ NPs can be recovered as a powder or as a suspension (Jamkhande et al. 2019). This approach offers several advantages, including its low cost and simplicity, as carbon dioxide or water can be used instead of organic solvents. Additionally, the synthesis is extremely fast, producing NPs in tens of seconds when compared to other conventional methods (Sankula et al. 2014; Philippot et al. 2014). However, it requires high pressure and critical temperature for NP preparation, and can prove to be a barrier (Jamkhande et al. 2019).

#### Sonochemical synthesis

Sonochemical synthesis utilizes ultrasound waves for the synthesis of NPs under mild conditions (Jajko-Liberka et al. 2025). Ultrasonication has proven to be an effective method for the generation of NPs with attractive properties in a short period of reaction time (Guo et al. 2011). The enhanced effect of ultrasonication is due to the acoustic cavitation phenomena, which is characterized by the rapid formation, growth, and collapse of bubbles in liquid. The extremely high conditions inside the collapsing bubble, such as temperature (> 5000 K), pressure (> 20 MPa), and cooling rate (> 1010Ks^−1^) create unique properties, and are able to reduce metal ions to metal or metal oxide NPs(Guo et al. 2011; Gedanken et al. 2001; Chen et al. 2001). For TiO_2_ NP synthesis, the precursor solution is subjected to ultrasonic irradiation at frequencies ranging from 20–40 kHz, the duration of sonication can range from a few minutes to an hour, depending on the required properties and reactivity of the system (Najafidoust et al. 2026). Sonochemical synthesis is particularly advantageous due to its enhanced photocatalytic capabilities due to the method’s ability generate nanocrystalline or submicron-sized NPs. These NPs provide greater surface area, and more exposed active sites which are essential for photocatalysis (Najafidoust et al. 2026). Additionally, the method is relatively cheap, has ambient conditions, and avoids use of toxic compounds (Matyszczak et al. 2024). However, the primary concerns surrounding sonochemical synthesis is the reproducibility and scale-up of the process. This can lead to inconsistent product quality such as compromised thermal stability and durability (Najafidoust et al. 2026).

#### Laser ablation

In laser ablation, laser irradiation is used to create NPs. The solid target is placed under a thin layer and subjected to pulsed laser exposure. Commonly used lasers include neodymium-doped yttrium aluminum garnet (Nd:YAG) operating at 1064 nm, along with Ti:Sapphire lasers and copper vapor lasers (Simakin et al. 2004; Borkenstein and Borkenstein 2024). Irradiation of the target material leads to the breakdown of the solid material into NPs, which disperse into the surrounding liquid medium and form a stable colloidal solution. The duration and intensity of the laser determine the relative number of NPs formed. Variation in laser pulse duration, laser fluence, ablation time, and the surrounding liquid medium (with or without surfactants) affects efficiency of ablation as well as the characteristics of the NPs (Jamkhande et al. 2019). Advantages of laser ablation include its relatively simple and effective technique for production of a large number of NPs. Additionally, laser ablation allows for NPs to be formed without surfactants, and also allows for control over its properties (Simakin et al. 2004). However, prolonged laser ablation can result in the accumulation of NPs in the colloidal solution, which can block the laser path from reaching the target surface, leading to reduced ablation rates (Jamkhande et al. 2019; Ghorbani 2014).

#### Flame-spray pyrolysis

Flame-spray pyrolysis is a cost-effective method widely used for the large-scale commercial synthesis of NPs. In this method, titanium precursors are atomized through a two-fluid nozzle and combusted high temperatures, where rapid oxidation leads to NP formation within milliseconds and collected as a dry powder. A typical flame-spray pyrolysis reactor consists of three parts: a two-fluid nozzle for delivery of the precursor and atomization, a premixed or diffusion burner as an ignition source, and a downstream filtering system that aids in the collection of the NPs (Alhaleeb and Machin 2020). Configuration of the flame spray pyrolysis system affects both system performance as well as characteristics of the NPs. The diameter of the precursor droplets and residence time of particles in the flame affects the physiological and chemical properties of the NP (Alhaleeb and Machin 2020; Teoh et al. 2010). The major advantages of flame step pyrolysis include short manufacturing times, high reproducibility, and the absence of liquid byproducts that are difficult to dispose of. It is also especially relevant in the healthcare industry as it enables precise control over the physiology of NPs, while being chemically pure (Li et al. 2026).

An important goal in TiO_2_ NP synthesis is producing particles in the quantum dot size range (2–10 nm) (Magaña et al. 2026). At this size NPs were found to be removed quickly (under 24 h) through the kidney and excreted through renal mechanisms, therefore reducing NP accumulation and possible toxicity (Longmire et al. 2008; Gustafson et al. 2015). Recently, sonochemical synthesis demonstrated the ability to form TiO_2_ quantum dots in the range of 2–4 nm at the frequency range of 20 kHz-2 MHz in an environmentally friendly manner (Magaña et al. 2026). However, reaching this size range at a consistent, cost-effective scale remains challenging.

Once the NPs have been synthesized, they can be used in a variety of fields, including environmental remediation, photocatalysis and antimicrobial coatings (Altammar 2023). In this review, we will focus on their use in anticancer treatments as a drug delivery agent, highlighting the underlying mechanism behind their action in the following section.

## TiO_2_ in anticancer therapy

### Nanoparticle-mediated drug release and delivery

Conventional drug delivery systems, administered through oral, pulmonary, parenteral, transdermal routes and intravenous pathways are used for the treatment of various diseases. However, they often suffer from several limitations, including instability, risk of displacement, uncontrolled release, slow absorption, enzymatic degradation, and side effects such as irritation and pain (Sultana et al. 2022). The advancement of NP technology has made them a promising candidate for controlled drug delivery systems. NPs have been found to improve the efficiency of therapeutic drugs by increasing their half-life and solubility, thereby enabling sustained and controlled drug release. Additionally, stimuli-responsive NPs have also been found to lower toxicity and control biodistribution (Dang and Guan 2020). Among the different NPs explored for drug delivery, TiO_2_ NPs are particularly useful due to their high biocompatibility, their ability to tune drug release, low toxicity, excellent photocatalytic activity, and overall stability (Ghareeb et al. 2024; Jafari et al. 2020). Compared to other inorganic nanomaterials, such as graphene, manganese oxide, and silica, TiO_2_ NPs offer distinct advantages. These include controllable size and morphology, easy functionalization, stable physiochemical properties, and high drug loading capacity (Zuo et al. 2024). However, challenges remain, such as the photocatalytic degradation of surface ligands under light exposure (Borah et al. 2021) and off-target organ accumulation (Shi et al. 2013). The following sections examine how surface and polymer modifications, molecular interactions, stimuli-responsive mechanisms, and biomimetic strategies have been developed to overcome these limitations and further refine drug release, stability, biocompatibility, and efficacy in TiO2 NP-based drug delivery.

#### Surface and polymer modifications

The morphology and pore structure of the NPs have a crucial impact on the sustained release of drugs. Thus, it is key to design TiO_2_ NPs with relatively small pores, as it enables a longer period of drug release which is essential for therapeutic efficacy. Mesoporous titanium dioxide (MTN) is a class of TiO_2_ NPs with an ordered porous framework, with pore sizes ranging from 2–50 nm. It is particularly effective in drug release modulation due to its modifiable porosity and large surface area (Khoz et al. 2024). When tested against zirconium dioxide over a 28-day period with diffusion-based release and amoxicillin as the model drug, MTN proved to have a better release rate due to its porous microstructure. Porous microstructures are good candidates for drug delivery as they can store medication in their pores, thereby increasing their capacity to release drugs (Mabrouk et al. 2022). Similarly, hollow NPs with controlled pore size through hydrothermal synthesis were also tested. The higher surface area allowed for better loading of the drug, while also making it easier for the drug to enter the active site (Cui et al. 2021).

Polymer modification of TiO_2_ NPs plays a key role in improving stability, biocompatibility, and drug loading while simultaneously allowing for targeted drug delivery. Polymer modification on the TiO_2_ NP surface increases the diffusion distance and introduces steric hindrance, resulting in sustained drug release. A popular polymer modifier is polydopamine, which was found to increase the thermal stability of TiO_2_ NPs, which is important as thermal stability ensures that the NPs retain their structural integrity and drug loading capacity in normal physiological and elevated temperatures (Dong et al. 2021). Polydopamines’ self-polymerization ability was also found to enhance the drug loading and prolong drug release in TiO_2_ nanotubes (Li et al. 2020), which were used due to their excellent biocompatibility (Wu et al. 2024). Polypyrrole-coated mesoporous TiO_2_ NPs provided for superior drug loading capacity, which allowed for the co-delivery of aspirin prodrugs along with doxorubicin. This modification enabled simultaneous activation of the prodrug and sustained drug release (Hajareh Haghighi et al. 2023).

#### Molecular interactions

Taking advantage of strong interaction forces like hydrophobic or electrostatic interactions could also allow for sustained drug release. MTN films loaded with ibuprofen, an anti-inflammatory drug used as a model molecule to demonstrate the encapsulation and elution behavior of MTN, and exhibited sustained drug release for 30 h due to the joint effect of curved pores and the hydrogen bonding interactions between ibuprofen, and the TiO_2_ films (Chao et al. 2012). The anticancer drug, daunorubicin, has three potential binding sites which allow for the formation of TiO_2_ complexes. To facilitate electrostatic interaction, the TiO_2_ surface is pretreated to become negatively charged using an aqueous solution with pH 7.4. Under these conditions the daunorubicin self-binds to the TiO_2_ surface using electrostatic interactions. This allows for the sustained release and retention time of the drug in the bloodstream (Chen and Zhang 2012).

#### Stimuli-responsive and targeted drug release

Monodispersed, organically-modified TiO_2_ NPs allowed for better drug loading along with more sustained drug release in environments with an acidic pH, such as the tumor microenvironment (TME). The TME is characterized by acidic pH, hypoxia, and redox imbalance, all of which play a crucial role in the designing and activation of TiO_2_ NPs. These features can aid in enabling and enhancing stimuli-responsive drug release, which allows for controlled and site-specific delivery within tumor tissues while reducing the risk of release in physiological conditions, thereby improving therapeutic efficacy (Sethi and Roy 2015; Uthaman et al. 2018). Pulit-Prociak et al., modified TiO_2_ NPs with glutathione, and the increased concentration of glutathione in the tumor cells initiated the breakdown of glutathione-TiO_2_ NP bonds, and enabled sustained drug release. This allows for targeted drug release under hypoxic conditions, enhancing specificity to target cancerous tissues (Bhullar et al. 2022a). Similarly, the Warburg effect, defined as the occurrence of aerobic glycolysis and lactic acid fermentation in tumor cells results in the formation of an acidic extracellular environment. This has led to further development of pH-responsive drug delivery systems for controlled drug release (Murugan et al. 2021). In one such study by Han et al., TiO_2_ NPs with narrow size distribution were synthesized with a poly(acrylic acid)-calcium phosphate (PAA-CaP) composite layer for breast cancer treatment. The PAA-CaP layer allows for dual functionality, as the composite layer is pH-responsive, enabling drug delivery, while the TiO_2_ core generates reactive oxygen species (ROS) through photoinduction. Results showed faster drug release in the acidic environment of the TME, along with increased cytotoxicity towards MCF-7 breast cancer cell lines (Han et al. 2022). Beyond pH-responsive mechanisms, external stimuli such as light are also important stimuli for controlled TiO_2_ NP drug release.

Light has emerged as one of the most promising external stimuli due to its ability to facilitate temporal and spatial control over the release of anticancer agents. Therefore, TiO_2_ NPs have attracted great attention as a photoactive drug carrier (Jafari et al. 2020). The photocatalytic properties of TiO_2_ have been shown to induce cytotoxic effects that can effectively kill tumor cells (Lagopati et al 2021). For instance, porous TiO_2_ NPs have been used to deliver paclitaxel, where drug release is triggered by ultraviolet (UV) radiation. When the same system was modified with polyethyleneimine (PEI), an anticancer agent, it showed greater potential for targeting tumors and drug release (Wang et al. 2015). To overcome the limited activity of TiO_2_ NPs under deeper penetrating wavelengths, an MTN-based system for near-infrared light-triggered drug delivery was developed by loading doxorubicin and capping the NPs with hyaluronic acid (HA). The HA coating allows the NPs to target CD44 receptors which are often overexpressed in cancer cells. It was observed that cell viability decreased at low drug doses, making it promising for cancer therapy (Ren et al. 2015). Similarly, colloidal TiO_2_ NPs were used as drug carriers for the light-controlled delivery of the ruthenium complex, which, on its own, exhibits lower toxicity to the melanoma cancer cell line. It was found that there was a faster release profile and greater cell death when stimulated with UV light as compared to red light (Nešić et al. 2017). Finally, Al-Nemwari et al., designed a biodegradable polymeric NP with chitosan, which was loaded with methotrexate and functionalized with TiO_2_ NPs for breast cancer treatment. Drug release can be triggered by UV light while being dependent on the amount of TiO_2_ NPs fixed on the coating, and this system showed significant efficacy against MCF-7 breast cancer cells (Al-Nemrawi et al. 2022).

Building on these systems, recent research has focused on advanced approaches such as protein corona (PC) modulation, ligand functionalization, and exosome coating. These approaches help further refine the specificity, biocompatibility, and efficacy of TiO_2_ NP-based drug delivery systems, and are discussed in the following sections.

#### Advanced targeting and biomimetic strategies

When exposed to biological fluids, such as whole blood, plasma, serum proteins adsorb onto NP surfaces and form a PC. The PC alters the physiochemical properties of NPs such as its bioactivity, destination, stability, therapeutic efficacy, and safety of drug carriers (Bashiri et al. 2023; Xiao et al. 2022). The unique physiological properties of the TME, such as the acidic pH, can alter the stability and structure of the PC, which consequently affects their adsorption onto the surface of TiO_2_ NPs (Mayordomo et al 2025). A 2022 study showed that the binding of dopamine-functionalized TiO_2_ NPs was sensitive to the pH. As changes in pH were observed, the protonation state of dopamine ligands and protein residues were altered, affecting PC formation on the NP (Siani and Di Valentin 2022). Additionally, Lastra et al. investigated the composition PC of TiO_2_ NPs covered in dopamine in HeLa cervical cancer cells. This study revealed that heat shock protein 90 (HSP90) and poly(ADP-ribose) polymerase 1, two cancer-related proteins, played a major role in the composition of the PC. This finding was significant as the selective depletion of these proteins onto the dopamine-coated TiO_2_ NPs suggests intracellular trafficking and interference with key pathways related to tumor survival (Omar Lastra, et al. 2019). Formation of the PC can also be used to enhance drug delivery. For example, adsorption of plasma proteins such as apoliproteins and albumins on NPs can aid immune evasion, prolonging circulation time of the NP (Li and Acta Materialia Inc 2021). Ultimately, significant knowledge gaps still remain. Studies mostly focus on hard PC, while soft PC remains largely unexplored, despite its biological relevance (Bashiri et al. 2023). Additionally, most in vitro studies overlook key in vivo biological processes such as circulation time, exposure of NPs to cells and biological fluids, and other pathological conditions, all of which affect PC composition and nature (Xiao et al. 2022). Furthermore, adsorption of proteins onto the NP surface within the PC can induce structural changes in proteins, which can expose epitopes which can activate unwanted immune responses (Mayordomo et al 2025). Understanding and modulating PC composition on TiO_2_ NPs, especially in tumor conditions, can thus enhance targeted drug delivery and optimize cancer treatment.

Recent advances in targeted nano delivery systems have begun to take advantage of ligand functionalization to further refine targeted delivery and drug release technology. Guo et al., developed an MTN system functionalized with HA and ADH-1 (a cyclic pentapeptide), and demonstrated enhanced delivery of doxorubicin to cancer cells. Here, HA acted as a targeting ligand to achieve targeted delivery towards CD44 overexpressing tumor cells, while simultaneously acting as a cross-linking molecule to conjugate ADH-1 on the surface of the MTN. ADH-1 aided in blocking the N-cadherin, disrupting EMT and making the tumor drug-sensitive. Lastly, the doxorubicin in combination with MTN-produced ROS was able to effectively kill the tumor cells (Guo et al. 2018). Bhullar et al., synthesized magnetically guided TiO_2_ NPs by coating it with Autrin, an iron supplement conjugated with folic acid. Autrin was integrated here due to its tendency to target areas with an abundance of folate receptors. Owing to the acidic pH of the TME and folate receptors associated with cancerous cells, the iron salt-folic acid combination was found to be effective in treating tumor cells. It was observed that drug release was at its highest in acidic environments, and the NP also displayed sustained drug release (Bhullar et al. 2022b). Despite being promising, ligand-functionalized TiO_2_ NPs remain unexplored, largely due to the photocatalytic nature of TiO_2_, in which TiO_2_ catalyzes the degradation of organic ligands when the system is exposed to light. An illustrative example for this phenomenon is the degradation of oleylamine ligands on TiO_2_ within two hours of UV exposure, exacerbating the joint effect of the inherent instability of organic ligands when exposed to irradiation, and the photocatalytic activity of TiO_2_ NPs (Borah et al. 2021). To overcome this limitation, strategies such as surface passivation using organic or inorganic coatings (e.g. PEG or silica) have been developed to protect functionalized ligands from degradation. Silica shell formation particularly provides enhanced stability, biocompatibility, and prevents direct interaction between reactive species and surface-bound molecules (Hajareh Haghighi et al. 2023). Additionally, doping and composite formation can control photocatalytic activity and modulate ROS generation, which reduces the chance of unintended degradation of organic ligands (Nasirian and Mehrvar 2016). These approaches preserve both the functionality and structure of the ligand.

Exosomes are small vesicles that are excreted by cells, akin to tumor cells, and have garnered attention as potential agents for tumor-specific drug delivery. Due to their intrinsic nature, exosomes can avoid natural barriers, and prolong circulation time in vivo. They also exhibit low toxicity and low immunogenicity, while having highly specific cell targeting properties (Huyan et al. 2020). A novel nanoplatform mimicking exosomes was recently developed by Z.Han et al., using oxygen deficient TiO_2_ (TiO_2-x_) coated with exosome membranes that were derived from glioblastoma cells. TiO_2-x_ was used due to its PTT conversion efficiency, and ROS generation under near-infrared (NIR) irradiation. In this system, doxorubicin was loaded into the TiO_2-x_ core, while the exosome coating enabled efficient penetration across the blood–brain barrier (BBB), and tumor specific accumulation, which is highly relevant for glioblastoma-specific therapy. Once irradiated by NIR, the nanoplatform triggered ROS production and localized heating, leading to controlled drug release and destruction of tumor cells (Han et al. 2025). Despite this, studies focused on exosome-coated TiO_2_ NPs are severely lacking, and require expansion on key areas, including scope of applications and specific limitations as well as advantages. This gap can be reasonably attributed to the complexity of exosome isolation and NP coating. Other critical challenges include as low exosome production as well as a lack of standardized protocols, must be addressed. Furthermore, unmodified exosomes often accumulate in off-target organs when studied in vivo*.* wherein off-target areas, they are promptly eliminated, limiting their utility and availability (Huyan et al. 2020; Han et al. 2025; Khidr et al. 2025). There are also questions regarding their safety, in terms of immunogenicity, biodistribution, and off-target toxicity due to exosomes differing in size, functionality, composition, and cellular heterogeneity (Khidr et al. 2025). These challenges and concerns regarding the use of exosomes in NP-based therapies must first be fully addressed before they can be fully implemented in clinical settings. Table 2 provides the different modification parameters for TiO_2_ NPs along with their effects on various cancers.

**Table 2:** Different modification parameters for TiO_2_ NPs and their effects on various cancers

Modification Parameter	Example	Effect of NP Properties	Cancer Model	Therapy	Observed Effect	Reference
Metal Doping	Cerium (Ce)-Doping	Improved visible-light absorption due to Ce^3^⁺/Ce^4^⁺ redox cycling	Retinoblastoma (Y73 cells)	PDT	Enhanced ROS generation and significant, dose-dependent reduction in cancer cell viability compared to undoped TiO₂	Kartha et al. 2022)
Non-Metal Doping	Carbon doping of TiO₂ (C-TiO₂)	Anatase phase, spherical 5–15 nm; bandgap narrowing; enhanced visible-light absorption in blue region	Cervical Cancer (HeLa cells)	PDT (blue light, 120 µW, 15 min)	C-TiO₂ + blue light reduced viability by 60%; ROS-mediated photokilling with autophagy as primary death pathway	Matijević et al. 2021)
Surface Functionalization	PEGylation of undoped and doped TiO₂ (Co, N, and Co + N) via sol–gel synthesis	Improved aqueous solubility and bioavailability; PEG coating enhanced cell permeability; metal/non-metal doping shifted photoactivation into visible/near infrared range	Cervical Cancer (HeLa cells)	PDT (UV and sunlight activation)	PEGylated undoped TiO₂ achieved ~ 75% cancer cell killing at 5.5 μg/mL under UV/sunlight; doped variants showed higher photoactivation but lower cytotoxicity, possibly due to reduced ROS or membrane permeability	Shah et al. 2019)
Ligand Conjugation	Glutamine-functionalized TiO₂ NP conjugated with thiosemicarbazone (TiO₂@Gln-TSC)	Glutamine enhances tumor cell uptake; TSC provides DNA synthesis inhibition potential	Hepatocellular Cancer (HepG2 cells)	Chemotherapy/ROS-mediated apoptosis	IC₅₀ 75 µg/mL in HepG2 vs. 210 µg/mL in HEK293; 27.3% apoptosis induction (vs. 2.8% control), 34.1% sub-G1 arrest; nuclear damage with chromatin fragmentation; higher selectivity for cancer cells due to glutamine targeting and combined ROS/TSC action	Shahmoradi et al. 2023)
Crystal Phase	Commercial TiO₂ NPs, size ~ 110–130 nm; dispersions of 100% anatase or anatase–rutile mixture (75:25); ultrasonication to reduce aggregation; UVA irradiation (350 nm, 20 min)	Pure anatase exhibited higher ROS generation; greater proapoptotic signaling than rutile-containing particles	MCF-7 (low invasive) and MDA-MB-468 (highly invasive) human breast cancer epithelial cells	Photodynamic therapy (UVA-activated	Selective apoptosis in MDA-MB-468 via Bax upregulation, caspase-mediated PARP cleavage, and DNA fragmentation; MCF-7 minimally affected	Lagopati et al. Jul. 2014)
Biomimetic Coating	Cancer cell membrane–coated hollow mesoporous TiO₂ nanoparticles loaded with hydroxychloroquine sulphate (CCM-HMTNPs/HCQ)	Hollow mesoporous TiO₂ core offers high surface area and pore volume for drug loading, HCQ inhibits autophagy and normalizes tumor vasculature; cancer cell membrane coating enables immune evasion, prolonged circulation, and homologous tumor targeting	Breast Cancer (MCF-7 Cells)	Sonodynamic therapy (US-activated) combined with autophagy inhibition	CCM coating improved tumor targeting and stability, HCQ blocked SDT-induced autophagy, alleviated tumor hypoxia, and sensitized tumors to SDT, enhanced ROS generation and tumor ablation without major organ toxicity	Feng et al. 2019)
Hybrid Composites	TiO₂–Au–PEG–Curcumin nanohybrid	TiO₂ core as sonosensitizer, Au nanoparticles reduce band gap and inhibit electron–hole recombination via SPR, PEG improves stability and circulation; curcumin adds intrinsic anticancer activity and enhances ROS generation	Cervical Cancer (HeLa Cells)	Sonodynamic therapy (US-activated)	Under US irradiation, IC₅₀ decreased from 122 to 38 μg/mL, significantly enhanced ROS generation, inhibited colony formation and migration, and disrupted spheroid growth, high stability over storage and repeated US cycles	Haghighi et al. 2024)
Morphology	Cancer cell membrane-coated carbon-doped TiO₂ hollow nanoshells (C-TiO_2_ HNSs)	Hollow nanoshell morphology provides large internal cavity for high tirapazamine drug loading and efficient ROS diffusion, carbon doping narrows band gap, improving sonodynamic performance, cancer cell membrane coating enhances homologous targeting, immune evasion, and greater circulation time	Colorectal Cancer (CT26 murine cells)	Combined sonodynamic therapy (US-activated) and hypoxia-activated chemotherapy	Under US irradiation, C-TiO₂ HNSs generated ROS and depleted oxygen to induce tumor hypoxia, activating tirapazamine’s cytotoxicity; synergistic SDT + BRT enhanced tumor ablation, prolonged survival, and showed good biosafety without major organ toxicity	Ning et al. 2022)

### Nanoparticles as therapeutic agents

Beyond drug delivery, TiO_2_ NPs are also utilized in anticancer therapies. TiO_2_ NPs have been used in PDT and PTT. Recent studies have also explored their use in combination with sonodynamic therapy and immunotherapy. The role of TiO_2_ NPs in these therapies is discussed below.

However, we can also examine the role of TiO_2_ NPs as sole therapeutic agents. As highlighted in previous sections, these NPs primarily induce ROS generation, which ultimately leads to a reduction in cell viability, and apoptosis-adjacent morphological changes, as observed in osteosarcoma and chondrosarcoma cell lines (Sha et al. 2013).Similar effects were observed in HCT116 colon cancer cell lines, and these effects directly correlate to the concentration, uptake of the NPs and time of exposure as well (Maddah et al. 2023). Another consistent effect observed here was a decrease in antioxidant enzyme activity, i.e., catalase. In a study conducted on hepatic cells (WRL-68), cell viability was found to be unaffected, although ROS generation and catalase activity were increased and decreased, respectively (Chibber 2017).Food-grade TiO_2_ NPs similarly induced ROS generation alongside alterations to epigenetic histone deacetylases in lung cells (A549), raising concerns regarding long-term exposure (Jayaram and Payne 2020). The ineffective nature of these NPs as sole therapeutic agents necessitates the usage of this nanoplatform as a combined therapeutic modality, as discussed subsequently.

#### Photodynamic therapy

PDT is a non-invasive therapeutic approach that uses photosensitizers alongside specific wavelengths to target damaged tissues. This induces ROS generation which leads to cell apoptosis (Li et al. 2024).When compared to traditional photosensitizers, metal-based NPs have greater extent of control during release and enhanced target specificity, slower degradation times, and longer cycle times. The most commonly used metallic NPs are zinc oxide (ZnO) NPs and TiO_2_ NPs (Sargazi et al. 2022). Recent studies have focused on the use of TiO_2_ NPs in PDT as these NPs tend to have a large surface area, are highly stable, and have optimal photocatalytic properties, which are crucial for efficient ROS production. Upon exposure to UV radiation, ROS are produced through the redox reactions occurring between oxygen and water molecules on the surface of the NPs (Paszko et al. 2011). These ROS are cytotoxic and play a central role in inducing cancer cell death. Despite the advantages of TiO_2_ NPs as a photosensitizer, certain disadvantages do exist, such as the minimal penetration ability of UV light (~ 10–100 µm) (Meinhardt et al. 2008), and the harmful nature of prolonged exposure to UV radiation, which can prove toxic to humans, resulting in visible effects such as photoaging, immunosuppression and photocarcinogenesis (Matsumura and Ananthaswamy 2004).

Thus, several studies have instead proposed visible light as an alternative for PDT treatment due to deeper tissue penetration and safer profile, i.e., minimal adverse effects in humans. However, TiO_2_ NPs are largely inactive under visible light due to their wide bandgap energy (~ 3.2 eV in anatase form). This issue can be counteracted by doping the NPs with nitrogen, which has been shown to allow the generation of controllable ROS and cell death in the visible light spectrum (Moosavi et al. 2016). Modifying TiO_2_ NPs with graphene also helps reduce the energy band gap, therefore improving TiO_2_ efficiency (Sargazi et al. 2022). Robeldo et al., modified TiO_2_ NPs with a coated peroxide group (Ti (OH)_4_), which lowered the energy band gap from 3.2 eV to 2.3 eV, hence allowing the peroxide coating to absorb visible light and exhibit photocatalytic activity equivalent to UV light. The peroxide coating also demonstrated its ability to produce OH^−^ radicals (ROS) after several cycles of photodegradation (Robeldo et al. 2022). A 2022 study constructed a hypoxia-adaptive nanocomposite, TiO_2_@Ru@siRNA, for the treatment of oral squamous cell carcinoma (OSCC). siRNA targeting is emerging as a powerful therapeutic tool in cancer treatment, as it enables the silencing of specific genes disrupting oncogenic pathways and inhibiting the growth and metastasis of the tumor(Charbe et al. 2020; Zhou et al. 2022). It was designed by pairing ruthenium (Ru) with TiO_2_ NPs before loading a siRNA-targeting molecule, hypoxia inducing factor-1α (HIF-1α). The nanocomposite was able to produce ROS under visible light irradiation, while also inducing pyroptosis, a form of programmed inflammatory cell death, in OSCC cells, activating cancer immune responses (Zhou et al. 2022).

#### Photothermal therapy

Nanomaterials used in PTT often exhibit high absorbance in the NIR range, which allows for conversion of laser energy into thermal energy. This thermal effect generated by the activated PTT agents induces necrosis, necroptosis, and apoptosis in tumor cells and tissues (Sargazi et al. 2022). When used in combination with PDT, this approach improves tumor destruction through ROS-mediated oxidative stress and heat-induced cytotoxicity. The shortcoming of TiO_2_ NPs in PTT is similar to those encountered with PDT. Particularly, poor absorption in infrared and visible light regions, and limited ROS or heat generation are key issues. However, these disadvantages can similarly be overcome through modification/doping of the NPs. Ou et al., used the arc-melting technique to synthesize a TiO_2_ based Magneli-phase Ti_8_O_15_ NPs. These NPs showed increased photothermal conversion efficiency, while lowering the innate toxicity of PTT. When irradiated with near infrared light at 808 nm for 5 min, they were effectively able to destroy the tumor cells (Ou et al. 2016). TiO_2_ NPs with polyethylene glycol have been found to exhibit increased water biocompatibility and dispersibility of the NPs, with the modified NPs showing improved efficacy in eliminating solid tumors (Behnam et al. 2018). Nie et al., synthesized Ag@TiO_2_ NPs, which proved to be a high-performance PTT agent with a photothermal conversion efficiency of 65% and strong absorption of NIR light. They were synthesized by coating Ag NPs with TiO_2_ coating through the sol–gel method, and, due to the oxide coating, high photothermal cytotoxicity was observed in B16-F10 murine melanoma cells in mice. When tested in subcutaneous melanoma tumor models, the modified NPs were injected into the tumor before being irradiated with a laser at 808 nm for 1 min, with the results showing a decrease in tumor volume (Nie et al. 2020). Ge et al., synthesized an asymmetric nanostructure, L-TiO_2_-GNR, by controlling the growth of TiO_2_ on one of its ends through a gold nanorod. The heated electrons that are generated due to irradiation under a laser at 808 nm are harnessed by the structure, which induces PDT and PTT at 48 °C; Additionally, hydrogen therapy is induced, where molecular hydrogen is released and this joint occurrence been found to reduce both oxidative stress and inflammation in the TME. This approach was shown to be effective in treating mice injected with breast cancer (Ge et al. 2022). However, despite its therapeutic efficacy, PTT usage is riddled with safety concerns regarding heat diffusion, where excessive or poorly localized thermal energy may damage surrounding healthy tissue (Wang et al. 2022). Therefore, precise control over irradiation and its NP delivery is essential in minimizing off-target effects.

#### Sonodynamic therapy

SDT is a non-invasive therapeutic alternative that offers greater tissue penetration (> 10 cm) when compared to traditional therapies such as PDT and PTT due to its non-radiative nature and low attenuation coefficient (You et al. 2016; Datta et al. 2024). Additionally, SDT is less reliant on oxygen when compared to therapies such as PDT, making it particularly effective in treating deep-rooted or hypoxic tumors (Jeong et al. 2024). The therapeutic mechanism of SDT primarily relies on sono-cavitation, a phenomenon in which ultrasound waves induce the formation, oscillation, and collapse of microscopic bubbles in tissues. This mechanical process produces high temperatures and pressures, triggering ROS production. These ROS can damage cancer cells, and directly induce necrosis and apoptosis, while indirectly damaging vessels and inhibiting neovascularization of tumor tissues. An important part of this process is the incorporation of sonosensitizers, which are compounds (such as NPs) that increase ROS production when exposed to ultrasound stimulation (Hutton et al. 2024). In a notable study, You et al., prepared a hydrophilized TiO_2_ NP (HTIO_2_ NP) that was modified with the hydrophilic polymer carboxymethyl dextran to improve circulation, which improved tumor targeting (You et al. 2016; Thambi et al. 2014). HTiO_2_ NP-based SDT generated high levels of ROS both in vitro and in vivo*,* leading to tumor microvascular damage, while sustaining the expression of cytokines linked with inflammation(You et al. 2016). Building on the potential of TiO_2_-based sonosensitizers, combination therapies have also shown improved therapeutic efficiency. For example, one approach involved a combination of doxorubicin-loaded silk fibroin NPs with copper (Cu)-doped TiO_2_ NPs for local chemo-sonodynamic targeting breast cancer. Silk fibroin was chosen as a drug carrier due to its excellent biocompatibility, biodegradability, and sustained drug release at the site of the tumor. Meanwhile, doping of the TiO_2_ NPs with Cu enhanced ROS generation by narrowing the energy bandgap and increasing electron–hole separation when exposed ultrasound radiation. The dual platform enabled a synergistic therapeutic effect, where SDT-induced ROS, together with doxorubicin cytotoxicity, achieved a tumor inhibition rate of 83.65% in vivo (Maghsoudian et al. 2025). Despite several advances, the efficiency of SDT is still restricted by the rarity of viable sonosensitizers and complex TME. To deal with these limitations, TiO_2_-Au Janus NPs functionalized with the glutamine metabolism blocker 6-diazo-5-oxo-L-norleucin (DON) were developed as a sono-metabolic therapeutic platform. The Janus structure allows for greater generation of ROS through the increasing separation of charges between TiO_2_ and Au under ultrasound radiation. Simultaneously, DON is delivered to the tumor site, which improves immune response and disrupts tumor redox balance. This approach helps to remodel the immunosuppressive TME while also improving outcomes of therapy (Zheng et al. 2025).

#### Immunotherapy

Cancer immunotherapy has gained significant attention over the last few decades as a promising strategy for therapy. Metallic NPs show particular promise because of their tendency to migrate towards the spleen and lymph organs, where they interact with the resident immune cells, making them good candidates for delivery of immunotherapeutic drugs. Additionally, their potential for co-application with optical and heat based therapeutic methods such as PTT and PDT proves its advantages over other NPs (2018). For instance, Hesemans et al., formulated Cu doped TiO_2_ NPs (Cu-TiO_2_ NPs), the TiO_2_ NPs to ensure the controlled release of Cu^2+^ ions and Cu^+^ ions, which are known to induce immunogenic cell death and enhance antitumor immune response. The NPs promoted the release of heat shock protein 70 (HSP70), a danger-associated molecular pattern (DAMP), that activates immune surveillance mechanisms. Furthermore, the Cu-TiO_2_ nanocomplex triggered the activation of the NLRP3 inflammasomes, and promoted dendritic cell maturation, both of which are crucial for initiating tumor-infiltrating lymphocytes response (Hesemans et al. 2023). Another study utilized V-domain immunoglobulin suppressor of T-cell activation (VISTA) antibody-loaded Fe_3_O_4_@TiO_2_ NPs to combine SDT with immune checkpoint inhibition. The magnetic core, Fe_3_O_4_, provides enhanced imaging capability while TiO_2_ functions as a sonosensitizer to generate ROS, leading to oxidative tumor damage. The loaded VISTA antibody blocked the VISTA immune checkpoint pathway, often upregulated in the TME to suppress T-cell function. By inhibiting this pathway, the immunosuppressive TME is remodeled, which improves therapeutic efficiency (Hong et al. 2024). Another innovative approach used a TiO_2_ nanosensitizer (TiO_2_@CaP), which was designed to be activated in the TME. The TiO_2_ NP acts as a sonosensitizer for ROS production, and is coated with CaP, which breaks down as it enters an acidic environment. Once in the acidic environment, the TiO_2_@CaP entity is broken down into TiO_2_ and Ca^2+^ ions, which stimulates increased generation of ROS leading to overloading of Ca^2+^ ions. This process increased apoptosis and immunogenic cell death in the tumor cells. Additionally, when combined with anti-PD1, the system brings about an antitumor immune response, which results in the suppression of primary tumors targeted by SDT and distant tumors (Tan et al. 2021). However, NP-based immunotherapy may also pose risks related to immunotoxicity. Intravenous administration of NP drug delivery systems can activate pro-inflammatory immune responses, which can result in systemic side effects such as emphysema, lung fibrosis and cardiovascular diseases (Hofer et al. 2022).

Table 3 details further applications of TiO_2_ NPs cancer therapy.

**Table 3:** Anticancer applications of TiO_2_ NPs in various cancers

*Stimulus Based Treatment*						
**Cancer**	**Stimulus**	**Treatment Method**	**Treatment Agents**	**Dosage**	**Observed Effect**	**Reference**
Human Burkitt Lymphoma (Daudi Cell line)	High-energy shockwaves	Shockwave-triggered mechanical damage via NPs	Amorphous-titania propyl-amine functionalized NPs	Variable NP concentrations, single vs multiple shockwaves	Significant reduction in cancer cell viability upon shockwave stimulation	Vighetto et al. 2021)
Murine Melanoma	Laser excitation	PTT induced hyperthermia via NPs	PEGylated TiO_2_ NPs	1 mg/mL of TiO_2_ NP, with irradiation at 808 nm, intensity 2W/cm^2^ for seven minutes	Were found to induce 70% necrosis of tumor	Behnam et al. 2018)
Pancreatic Cancer (CD133^+^ cancer stem–like cells)	Near-infrared light (PTT)	MRI-guided photothermal therapy targeting CD133^+^ cancer stem-like cells	Black TiO₂-based nanoprobes (MRI-visible photothermal agents; CSC-targeting if functionalized)	150ug/mL	Showed improved PTT efficacy under laser irradiation	Wang et al. 2018)
Breast Cancer (MCF-7 Cells)	ROS-mediated cytotoxicity via Zn doping	Induction of oxidative stress through band gap tuning and particle size reduction	Zn-doped TiO₂ NPs (1–10 at wt% Zn)	50,100, and 200 ug/mL	Was found to induce greater cytotoxicity and oxidative stress in the cell line	Ahamed et al. 2016)
Cervical Cancer (HeLa Cells)	Visible Light	Photodynamic therapy via mutual sensitization — TiO₂ NPs extend HA photosensitization into visible range; GO carrier undergoes ROS-induced self-degradation, helping system metabolization	Hypocrellin A (HA) + TiO₂ NPs on graphene oxide (HA–TiO₂–GO)	1.1 mg/mL (HA-TiO_2_-GO)	Study showed improved visible light response in TiO_2_, and ROS generation, efficacy of ROS generation also improved, studies also showed that GO was destroyed by singlet oxygen, which is helpful for metabolism of the system	Ding et al. 2016)
Cervical Cancer (HeLa and KB cells)	Visible Light	PDT induced ROS	N–TiO2–Pc (Nitrogen-doped TiO2 nanoparticles conjugated with aluminum phthalocyanine chloride tetrasulfonate)	4.7 µg/mL of N–TiO2–Pc, with illumination at 15 J/cm^2^	ROS production increased 2.6 times when compared to Pc alone, cellular intake also improved 6 times. The photokilling effect of the NP was much greater than Pc alone	Pan et al. 2015)
***Targeted Treatment***						
**Cancer**	**Target**	**Treatment Method**	**Treatment Agents**	**Dosage**	**Observed Effect**	**Reference**
Bladder Cancer (T24 cells)	Folate Receptors	Targeted NP therapy inducing apoptosis	Folic acid-coated TiO_2_ NPs	21.8 ± 1.9 µg/mL	16.63% apoptosis induction in vivo was observed	Hanna et al. 2023)
Epithelial Cell Carcinoma (A431 cells)	Epidermal growth factor receptor (EGFR)	Targeted NP therapy for PDD/PDT	EGF-ligated PEG-coated TiO₂ NPs	10 μg/mL for 24 h	Increased uptake of conjugated NPs in cancer cells, while decreasing tumor cell proliferation	Salama, et al. 2020)
Breast Cancer (MCF-7 cells)	Homologous targeting via cancer cell membrane coating & autophagy inhibition	Targeted SDT with autophagy regulation	Cancer cell membrane-coated hollow mesoporous TiO_2_ NPs loaded with hydroxychloroquine sulphate (HCQ)	20 mg/kg of TiO_2_ NPs with 50 mg/kg hydroxychloroquine sulphate (HCQ)	Increased SDT sensitivity by blocking autophagic flux, improving tumor oxygenation, and overcoming SDT resistance	Feng et al. 2019)
Cervical Cancer (HeLa cells)	Folate Receptors	Photodynamic therapy under solar/UV light	PEG-coated TiO₂ NPs and folic acid–conjugated TiO₂ NPs (doped and undoped)	5.5ug/mL	Was found to kill 75% of cervical cancer cells	Shah et al. 2019)
Breast Cancer (MDA-MB-231 and Hs578T cells)	Epidermal growth factor receptor signaling pathway	NP-mediated receptor–ligand disruption	Titanium dioxide (TiO₂) NPs	10,50,100 ug/mL	Caused cytotoxic effect in breast cancer by interfering with the EGFR signaling pathway	Kim et al. 2019)
Osteosarcoma (MG63 cells)	Folate Receptors	Ligand-mediated targeting via folate receptor	Folic acid–tagged TiO₂ nanoparticles	N/A	Showed significantly higher generation of apoptosis in cancer cells(38% vs 16%), greater ROS generation	wei Ai et al. 2017)
***Combination Therapies***						
**Cancer**	**Role of TiO**_**2**_** NPs**	**Treatment Method**	**Treatment Agents**	**Dosage**	**Observed Effect**	**Reference**
Murine Melanoma	Sensitization to chemotherapy	Chemotherapy + NP-assisted therapy	TiO_2_ NPs + Cisplatin	Variable TiO_2_ NP concentration + variable cisplatin concentrations	Was found to inhibit cell proliferation and reduce tumor growth	Adibzadeh et al. 2021)
Breast Cancer (MCF-7 cells)	ROS generation via SDT and controlled release of AQ4N	SDT + hypoxia-specific chemotherapy	RBC membrane-coated TiO₂-based nanoparticles (mTNPs) loaded with AQ4N	25 mg/kg	Inhibited growth of tumor by 100% in mouse model	Li et al. 2021)
Hepatoma Cancer (Hepa 1–6 cells)	Sono sensitization for SDT + immune stimulation	SDT + checkpoint blockade immunotherapy (aPD-L1)	TiO₂–Ce6–CpG nanosonosensitizers + aPD-L1 antibody	100 µg/mL	Was found to Inhibit tumor growth and stimulate immune system	Lin et al. Mar. 2021)
Oral Squamous Cell Carcinoma (HSC-2 cells)	Sonosensitizer for ROS generation, enhances ultrasound-induced cytotoxicity	High-intensity focused ultrasound (HIFU) + TiO₂-mediated sonodynamic therapy	TiO₂ NPs + HIFU	0.01,0.03, and 0.06% (v/v) in vitro 10uL in vivo	Cell toxicity in tumor had direct correlation with conc. of NP and intensity of HIFU in vitro, in vivo results showed TiO_2_ penetration of the tumor cell cytoplasm	Moosavi Nejad et al. 2016)

## Cytotoxic actions of TiO_2_ NPs

While TiO_2_ NPs have demonstrated great potential in anticancer therapies, their mechanisms of cytotoxicity remain context-dependent. This section focuses on the different mechanisms used by TiO_2_ NPs to induce cytotoxicity.

One of the most well-known forms of cytotoxicity is ROS generation, which can play a dual role in tumorigenicity. Low levels of ROS (typically reported in the range of 5–50 nM up to 0.7 µm) results in activation of cell proliferation mechanisms, such as the nuclear factor κβ (NF-κβ) and mitogen-activated protein kinase (MAPK) pathways, leading to increased tumorigenicity (Chatterjee et al. 2024; Dröge 2002; Thannickal and Fanburg 2000). At higher concentrations of ROS generation (commonly reported around 1–3 µM), ROS induce apoptosis through activation of apoptosis signal-regulating kinase 1 (ASK1), and poly (ADP-ribose) polymerase (PARP), alongside mitochondrial damage. Autophagy is simultaneously induced through activation of BNIP3, AMP-activated protein kinase pathway (AMPK), and MAPK, while inactivating a key protease known as Autophagy-related 4 (ATG4) (Chatterjee et al. 2024; Villalpando-Rodriguez and Gibson 2021). ROS levels exceeding 3 µM often result in necrotic cell death. TiO_2_ NPs induce ROS generation mainly through photocatalytic activity, whereby exposure to UV or visible light radiation drives redox reactions between water and oxygen on the surface of the NP, thereby producing superoxide and hydroxyl radicals, which are subsequently used in secondary mechanisms such as PDT and PTT (Paszko et al. 2011; Chatterjee et al. 2024). This surge in ROS causes dysfunction of mitochondria, lipid peroxidation, DNA fragmentation, and caspase-dependent apoptosis. For example, a study involving hepatoma HepG2 showed that TiO_2_ NPs induced genotoxic stress through oxidative DNA damage, primarily due to excessive ROS generation (Petković et al. 2011). However, the variability of ROS generation across various mechanisms is difficult to compare due to differences in measurement techniques (Dikalov and Harrison 2014) and external dependencies such as NP dosage.

The ability of TiO_2_ NPs to interfere with the antioxidant defense system has also attracted great attention in recent years. Exposure to these NPs leads to a sharp increase in ROS production, especially superoxide anions (O_2_^−2^). Under normal conditions, antioxidant enzymes such as superoxide dismutase (SOD), catalase, and glutathione peroxide help in ROS balance. However, TiO_2_ NPs can interfere with these pathways, which creates a pro-oxidative state. This imbalance leads to cumulative oxidative stress, which, in turn, can cause DNA damage, mitochondrial dysregulation, and cell death. A study on osteoblastoma cells showed that the use of TiO_2_ NPs greatly increased the production of superoxides while inhibiting the function of SOD and GPx, which generates excess ROS, and leads to cell death (Niska et al. 2015).

In addition to oxidative mechanisms, TiO_2_ NPs have also been found to induce cytotoxicity through alteration of important intracellular pathways, such as the epidermal growth factor receptor (EGFR), p53, and Akt. A recent study demonstrated that TiO_2_ NPs inhibit EGFR phosphorylation in breast cancer, disrupting downstream PI3K/Akt signaling, along with pathways key for cell growth and apoptosis prevention. Suppression of the Akt pathway resulted in reduced in Bcl-2 expression and activation of pro-apoptotic factors such as Bax and caspase 9, which trigger apoptotic cascades. Simultaneously, TiO_2_ NPs were found to upregulate p53, a tumor suppressor, which leads to the accumulation of p21, and growth arrest and DNA damage-inducible protein 45 (GADD45), facilitating apoptosis (Kim et al. 2019).

**Fig. 1:**
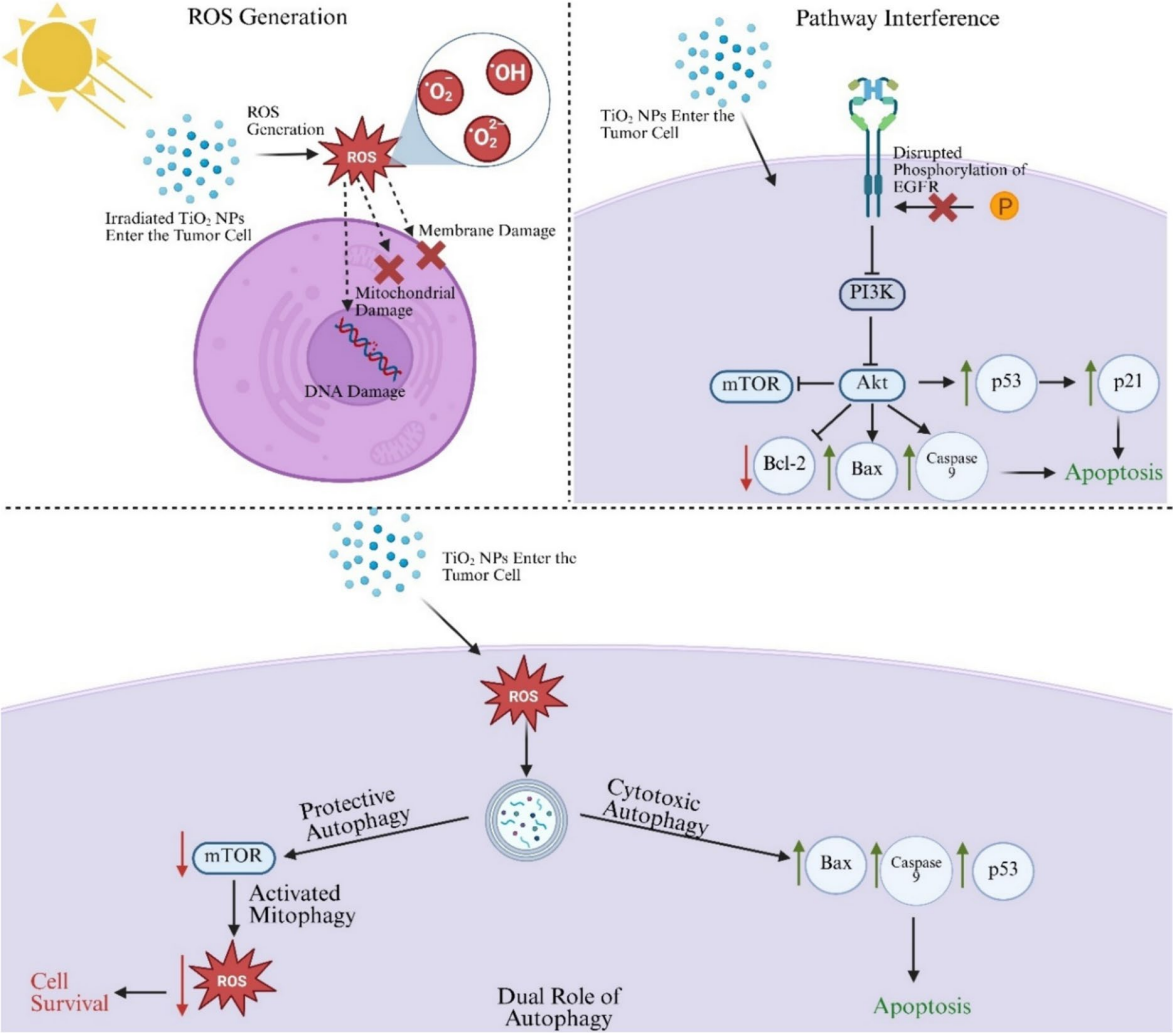
Cytotoxic mechanisms of TiO_2_ NPs (Created with Biorender) TiO_2 _NPs produces cytotoxicity through various mechanisms. **A** ROS generation: When irradiated, TiO_2_ NPs produces ROS in the form of superoxide anions, hydroxyl radicals, and singlet oxygen that can damage membranes and mitochondria, while leading to fragmentation of DNA, contributing to tumor cell death. **B** Pathway interference: These NPs can also interrupt EGFR phosphorylation, which results in PI3K/Akt signaling axis inhibition. Subsequently, this causes decreased Bcl-2 expression, Bax and Caspase-9 activation, and elevation of p53 which leads to apoptosis in cancer cells. **C** Dual role of autophagy: TiO_2_ NPs are known to cause autophagy through generation of ROS and inhibition of the mTOR complex. Autophagy can produce a protective effect through mitophagy and clearance of ROS which helps in survival of tumor cells. It can also shift towards a cytotoxic role when activated excessively, leading to Bax, Caspase-9, and p53 activation, leading to apoptosis thereby reducing cancer cell survival

Recent evidence suggests that NPs have the ability to modulate autophagy. Autophagy is the catabolic process by which endogenous and foreign materials are taken up by membrane vesicles and degraded upon fusion of said membrane vesicles with lysosomes (Peynshaert et al. 2014). While NPs have been found to be able to exploit autophagy for therapeutic outcomes, autophagy can also be triggered as a survival response from cells against NP-mediated toxicity (Florance et al. 2024). In gastric cancer cells, TiO_2_ NPs were shown to enhance chemotherapeutic efficiency of 5-flouroacil by blocking autophagic flux, thereby reducing the cells’ ability to trigger survival responses such as cryoprotective self-repair. This blockade results in the accumulation of damaged organelles, leading to the activation of stress signals, and shifting the cells toward apoptosis (Azimee et al. 2020). Conversely, in leukemia cells, photo-activated nitrogen-doped TiO_2_ NPs (N-TiO_2_ NPs) were found to induce the generation of ROS. These ROS function as cellular stress signals, particularly towards the mitochondria and endoplasmic reticulum, thereby activating autophagy. Depending on NP concentration, activation of autophagy can lead to terminal differentiation of tumor cells or apoptosis in tumor cells (Moosavi et al. 2016). These studies highlight the context-dependent role in determining tumor cell fate. The key cytotoxic mechanisms of TiO_2_ NPs, including ROS generation, pathway interference, and autophagy modulation have been illustrated in Fig. 1. Additionally, a comprehensive overview of TiO_2_ NP-induced cytotoxicity, highlighting its therapeutic benefit and associated risks are present in Table 4.

**Table 4:** Dual-role of TiO_2_ NP-induced cytotoxicity

Category	Therapeutic Effect	Associated Risks	References
ROS Generation	Induces apoptosis, autophagy, and necrosis via oxidative stress	Low ROS promotes tumorigenicity, variability in measurement and dosage limits comparability	Chatterjee et al. 2024; Dikalov and Harrison 2014)
Dose Dependency	Enables controlled ROS-mediated cytotoxicity	Inconsistent outcomes, low doses may promote proliferation of cancer cells	Chatterjee et al. 2024; Foglietta et al. 2023)
Measurement Variability	Enables detection and quantification of ROS in experiments	Differences in ROS detection methods can lead to misinterpretation due to probe leakage, non-specificity, and inability to differentiate between species of ROS	Zhao and Riediker 2014)
Long-Term Exposure	Sustained therapeutic effects	Chronic inflammation, DNA damage, oxidative stress generation	Alswady-Hoff, et al. 2021)
Organ Accumulation	__	Altered cellular function, inflammation, cytotoxicity	Mohammadparast et al. 2026)
Genotoxicity	ROS-induced DNA damage contributes to death of tumor cells	DNA damage in healthy cells, potential mutagenesis	Petković et al. 2011; Kirkland et al 2022; Shi et al. 2022)
Signaling Pathway Interference	Inhibits EGFR/PI3K/Akt signaling, and promotes apoptosis through p53 activation	Activation of DNA damage pathways, leading to cellular stress	Kim et al. 2019; cB. Han,, et al. 2020)
Autophagy Modulation	Enhances chemotherapeutic efficiency and promotes apoptosis	Can promote tumor cell survival	Moosavi et al. 2016; Florance et al. 2024; Azimee et al. 2020)

## The case for nanomedicine: comparing conventional therapy and TiO_2_ NP therapy for cancer

Although chemotherapy and radiotherapy remain the standard of care for cancer treatment, their efficacy is limited by drug resistance, off-target cytotoxicity, lack of tumor specificity, and limited tumor penetration (Zafar et al. 2025). These shortcomings have led to the development of alternative treatments such as nanoparticle-based systems like TiO_2_ NPs, which enable more targeted and possibly less toxic approaches. Chemotherapeutic drugs primarily target rapidly dividing cancerous cells, but can also attack healthy cells that replicate quickly, such as hair follicles, bone marrow, and the gastrointestinal tract due to the low specificity of chemotherapeutic agents. This often leads to harmful consequences for organs and organ systems, causing serious side effects and ultimately resulting in organ toxicity, which forces clinicians to use lower dosages and shorter treatments, reducing treatment efficiency (Rashidi et al. 2024). TiO_2_ NPs offer a more targeted approach. These NPs can be designed to accumulate in selective tumor tissue through passive mechanisms such as the enhanced permeability and retention (EPR) effect (Belyaev et al. 2025), or active targeting, such as binding to surface proteins that are overexpressed on tumor cells (Bazak et al. 2015). Beyond targeting tumor cells, TiO_2_ NPs also have photocatalytic properties that permit the implementation of light-controlled cytotoxicity. Other activation inducers include thermal energy, which enables site specific therapeutic action. This minimizes off-target toxicity, a common feature of chemotherapy, thus making treatments more tolerable (Sargazi et al. 2022; Paszko et al. 2011).

**Fig. 2:**
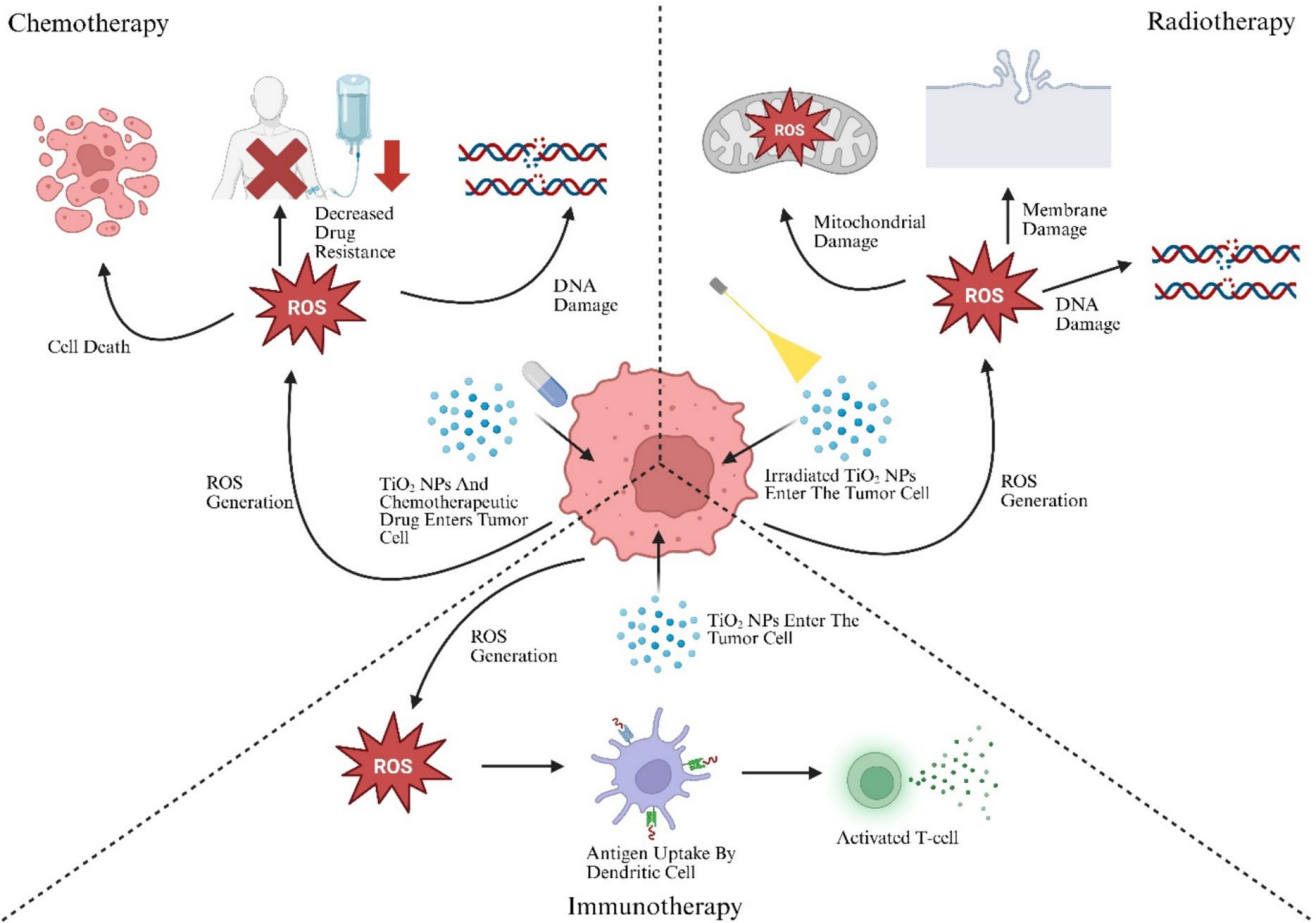
Enhancement of conventional cancer therapies using TiO_2_ NPs (Created using Biorender) TiO_2_ NPs may enhance the effectiveness of several forms of conventional therapy through ROS-dependent mechanisms, potentially increasing tumor cell susceptibility to treatments. **A** TiO_2_ NP-enhanced Chemotherapy: When combined with chemotherapeutic drugs, TiO_2_ NPs enter tumor cells and increase generation of ROS, leading to increased DNA damage and apoptosis, and may improve therapeutic efficacy (Adibzadeh et al. 2021; Azimee et al. 2020). **B** TiO_2_ NP-enhanced Radiotherapy: Upon irradiation, TiO_2_ NPs generate ROS within cancer cells, leading to mitochondrial dysfunction, DNA breaks, and damage to the membrane, thereby amplifying the cytotoxicity effects of radiotherapy (Nakayama et al. 2024). **C** TiO_2_ NP-enhanced Immunotherapy: ROS produced by TiO_2_ NPs induces immunogenic cell death leading to the release of tumor-associated antigens. These antigens are taken up by dendritic cells, triggering T-cell activation and enhancing anti-tumor immune responses, in a context dependent manner (Jia et al. 2018; Lian et al. 2025; Liu et al. 2024)

Radiotherapy, although localized, faces similar challenges to chemotherapy. Ionizing radiation damages the surrounding healthy tissues, and its efficiency is reduced in hypoxic tumor regions where production of ROS is affected by low oxygen levels. This results in incomplete tumor elimination, and could contribute to the recurrence and radio-resistance of the tumor (He et al. 2025). TiO_2_ NPs have also shown promise as radiosensitizers, as they can enhance the efficacy of radiation therapy in cancer treatment by increasing ROS production in hypoxic conditions and promoting DNA damage when used in combination with radiation therapy. This allows for more control of tumor sizes at lower radiation doses, which reduces the damage borne by healthy tissue (Nakayama et al. 2024). The role of TiO_2_ NPs in enhancing conventional cancer therapies is illustrated in Fig. 2.

### Usage of metal nanocomposites: why we use it and its impact

Incorporating metal ions into TiO_2_ NPs is an upcoming strategy to improve its long-term therapeutic outcomes. Despite its promise, pristine TiO_2_ NPs are limited by the need for large dosages and inconsistent effects across tumor subtypes (Moosavi et al. 2016; Gojznikar et al. 2022; Sedky et al. 2024). To address these limitations, TiO_2_ NPs are designed as nanocomposites with metal inclusions such as silver (Ag) and zinc (Zn), which helps enhance the therapeutic efficiency and anti-cancer effects across tumor subtypes.

For example, Ag-doped TiO_2_ NPs facilitated better ROS production, and therefore, greater pro-apoptotic activity when compared to undoped TiO_2_, which allows for the elimination of cancer cells at lowered dosages. Better ROS generation can be achieved through Ag doping, which lowers the bandgap energy required (3.32 eV vs 3.15 eV) for ROS generation through photocatalytic activity. Additionally, Ag-doped TiO_2_ NPs were found to induce cytotoxicity in cancer cells lines such as HepG2, A549, and MCF-7, while largely not affecting non-cancerous cells, providing evidence of their specificity (Ahamed et al. 2017). Likewise, Zn-doped TiO_2_ NPs were found to change the bandgap favorably which enables photocatalytic activity and generation of ROS, which led to glutathione depletion and apoptosis in multiple in MCF-7, A549, and HepG2 cells (Ahamed et al. 2016).

Besides cytotoxic enhancement, TiO_2_-Ag/Zn nanocomposites show greater antimicrobial effects, which cannot be achieved through traditional therapeutic options (Parvin et al. 2025). New studies show that microbial biofilms, especially ones formed by *Escherichia Coli* and *Staphylococcus Aureus*, are regularly found in chronic diseases. These biofilms are involved in driving tumorigenesis through inflammation and modulation of the immune system. They have also been found to be promote resistance to chemotherapy by shaping the local immune microenvironment (Montanari et al. 2025). Finally, TiO_2_-Ag/Zn nanocomposites were found to disrupt these biofilms, which reduces infection risk while suppressing the TME (Varma et al. 2023). TiO_2_ NPs also demonstrate a higher drug loading capacity to combat antimicrobial infections when compared to silica and copolymer platforms, as demonstrated in a 2024 study, where Ciprofloxacin, a common antimicrobial, was loaded in higher concentrations in titania systems compared to others (Sanattalab et al. 2024).

Despite their advantages, metal doping can prove to be toxic due to effects such as ion leaching, whereby dopant metal ions are gradually released from the NP under physiological conditions, possibly leading to off-target toxicity with repeated dosing. For example, Ag-doped TiO_2_ NPs were shown to cause greater histological legions, induce oxidative stress and apoptosis in the liver, gill, intestine and kidney tissues in zebrafish (*Danio rerio*) when compared to undoped TiO_2_ NPs (Mahjoubian et al. 2021). Furthermore, systemic toxicity beyond the site of administration was also reported, with Fe-doped TiO_2_ nanorods inducing pulmonary inflammation, hepatoxicity, and cardiovascular effects in vivo (Nemmar et al. 2011). These examples highlight the need for safety profiling of metal-doped TiO_2_ NPs before their implementation in clinical settings.

These findings highlight the potential of TiO_2_ NPs to address the limitations present in traditional therapeutic modalities by offering greater biocompatibility, therapeutic efficacy, and greater tumor specificity. The potential undesirable long-term effects of these NP systems require further characterization in vivo before human trials are conducted. These effects are examined in detail in the succeeding section.

## Long term effects of nanoparticle-mediated therapy

### Selective cytotoxicity and tumor subtype specificity

Despite the promising applications of TiO_2 _NPs, long-term application strategies remain complex. One of the primary advantages of TiO_2_ NPs is their selective cytotoxicity, as they have the ability to spare normal cells while inducing apoptosis and generating oxidative stress in cancer cells (Kukia et al. 2018). This selectivity is not consistent, with the effects heavily dependent on variation in NP uptake, intracellular trafficking, and additional inconsistency in efficiency across tumor subtypes. For example, green-synthesized TiO_2_ NPs have a half-maximal inhibitory concentration (IC_50_) of 1.73 µg/mL in MCF-7 breast cancer cells, and 4.79 µg/mL in A-549 lung cancer cells. Meanwhile, the IC_50_ of non-cancerous MCF10A cells was 15.17 µg/mL. The selectivity index (SI) is a parameter that quantifies the selectivity of a NP targeting cancer cells over healthy cells, and this parameter stood at 8.77 for breast cancer, while being 3.17 for lung cancer in a comparative study. This disparity in IC_50_ could be due to greater cytotoxicity observed in MCF-7 cells when compared to A-549 cells, suggesting greater efficiency of TiO_2_ NP-based treatments in breast tumor subtypes. MCF-7 cells showed strong activation of pro-apoptotic Bax and Bak genes (7.17 and 11.62-fold respectively), both of which drive programmed cell death. In contrast, A-549 cells showed weaker activation of the same genes (2.01 and 3.21-fold), and instead predominantly underwent necrotic cell death, with 13.62% of the population in the necrotic phase compared to 5.2% of MCF-7 cells (Sedky et al. 2024). This difference likely reflects how effectively the cell types respond to the cell death signal activated by TiO_2_ NPs, as indicated by the different expression levels of the Bax and Bak genes in the two cell lines.

### Long-term safety concerns

#### Dosage control and tumorigenicity

A major limitation of TiO_2_ NPs is the occurrence of potential side effects as a result of the high doses required for effective cancer treatment. Prolonged or repeated exposure can also lead to unintended side effects, such as the induction of tumorigenicity in non-cancerous tissues. In a landmark study by K.Onuma et al., mice were injected with QR-32 fibrosarcoma cells. Hydrophilic TiO_2_ NPs were found to alter the tissue microenvironment such that cells that were previously non-cancerous became cancerous. The change was linked to generation of ROS, which induces selective pressure, where sensitive cells were eliminated and ROS-resistant aggressive cells survived. These transformed cells showed greater release of factors such as prostaglandin E2 and transforming growth factor β, which helps in the progression of tumor growth (Onuma et al. 2009). These findings suggest that high dosages and continuous exposure to NP-based treatments may encourage malignancy, but further studies are needed to fully understand the mechanisms involved and their implications.

#### Metal accumulation and toxicity

Use of TiO_2_ NPs can result in the accumulation of the NPs in vital organs such as the liver, spleen, and kidneys, especially when the delivered dosages are high (Shi et al. 2013). These dosages can also cause TiO_2_ NPs to enter circulation and penetrate biological barriers such as the blood–brain barrier (BBB) and blood-placenta barrier where it can accumulate and lead to serious side effects (Shi et al. 2013; Xuan et al. 2023). An in vitro study found that TiO_2_ NPs can infiltrate the BBB with both long term and acute exposure. TiO_2_ NPs were found to reduce the concentration of tight junction proteins, while increasing cell–cell permeability, thereby causing inflammation in endothelial cells and inducing a state of increased oxidative stress. This indicated that use of these NPs in treatment could lead to neurological impairment (Brun et al. 2012). Furthermore, the continuous accumulation of TiO_2_ NPs has been attributed to genotoxic results, which includes DNA fragmentation, alterations in chromosomes, and formation of small, extra nuclei. These effects can be owed to the generation of ROS and interference with DNA repair pathways (Kirkland et al 2022; Shi et al. 2022), highlighting the caution needed when using TiO_2_ NPs in medicine.

An important factor in organ accumulation is NP size. TiO_2_ NPs larger than 8 nm cannot undergo renal filtration and clearance, due to it exceeding the threshold limits for glomerular filtration (Adhipandito et al. 2021). Consequently, larger NPs are taken up by mononuclear phagocytes and become concentrated in the liver, spleen, and blood. This uptake may paint a misleading picture when assessing toxicity, as cells in other tissues may not seem affected; however, it is not because the NPs are safe. It is due to larger NPs never reaching other cells due to their large size. In comparison, smaller TiO_2_ NPs spread widely throughout the body, thus interacting with a larger number of cells (Manuja et al 2021). Notably, NPs in the 40–50 nm ranged are internalized by receptor-mediated endocytosis, which allows them to reach tissues not generally accessible by larger NPs. Evidence also suggests that larger NPs still reside in the clearance organs, with limited clearance (Gustafson et al. 2015). This is especially concerning, given that TiO_2_ is frequently seen as a food additive, named E171. E171 is a white powder, where one-third of the particles fall in the NP range (< 100 nm), and studies have shown dose-dependent toxicity in organs, such as the liver, while TiO_2_ has been associated with inflammation, structural and functional modifications in the liver, and cell death (Khan et al. 2025).

#### Disruption of normal biological activity

TiO_2_ NPs have been found to interrupt regular biological activity, specifically through inflammation and oxidative stress mechanisms. These effects may lead to long term physiological damage, thereby affecting the safety of TiO_2_ NP-based treatments. One study also showed that TiO_2_ NPs induced sustained inflammatory response, which is defined by the upregulation of cytokines such as interleukin-6 (IL-6) and tumor necrosis factor α (TNF α). It was also found to encourage immune cell infiltration, in lung epithelial cells within the chorioallontoic membrane. This response promoted irregular proliferation alongside the breakdown of the extracellular matrix (Medina-Reyes et al. 2015). In another study, TiO_2_ NPs were found to be involved in the disruption of the epithelial barrier and immune dysregulation in the gastrointestinal tract. TiO_2_ exposure in the gut barrier compromised its integrity and changed the composition of the gut microbiota, a phenomenon which has been found to be associated with inflammation and immune activation. However, coating TiO_2_ NPs with a glutenin-based PC reduced these effects, restoring gut function and avoiding inflammation (Mi et al. 2024).

These long-term effects are closely linked to the cytotoxic mechanisms, which are discussed in Table 4.

### Clinical translation and regulatory barriers

Direct clinical evidence for the effects of long-term therapeutic use of TiO_2_ NPs on humans is sparse. Most studies focus on topical or oral exposure rather than cancer-focused therapeutic trials. For example, several studies have explored the role of TiO_2_ NPs in dermal penetration, with results showing that TiO_2_ NPs did not penetrate intact human skin, even when the substratum corneum was damaged (Sadrieh et al. 2010; Dréno et al. 2019). However, one trial involving older volunteers (ages 59–82) reported trace Ti accumulation in the superficial epidermal layers; however, toxicity was not observed. Inhalation studies have also demonstrated the possibility of accumulation in the lungs (21 nm) at high dosages in rats after 42 days of exposure, and it was also found that 28 days after exposure, TiO_2_ NPs were able to reach extrapulmonary tissues such as the kidney and liver (Shi et al. 2013). When ingested orally, TiO_2_ NPs remain in the major organs for an extended amount of time and are eventually excreted through stool. Absorbed TiO_2_ NPs often accumulate in major organs such as the kidneys, liver, spleen, and lungs and can cause possible nephrotoxicity and liver damage (Rashid et al. 2021). From a regulatory standpoint, several agencies have raised concerns regarding the safety of TiO_2_ NPs. The European Food Authority (EFA) banned TiO_2_ as a food additive in 2021, citing genotoxicity from chronic exposure (Dand et al. 2025). Additionally, the International Agency for Research on Cancer (IARC) has deemed it “possibly carcinogenic to humans”, while the National Institute of Occupational Safety and Health (NIOSH) has classified it as an occupational carcinogen (Skocaj et al. 2011). Furthermore, evaluation by the European Medicines Agency (EMA) have highlighted challenges in replacing TiO_2_ in medicinal products, due to a limited alternatives and potential impact on the safety, efficacy, and quality of the medicinal product if alternates were used. Several mitigation strategies discussed earlier, such as surface modification, targeted functionalization, and controlled drug-delivery systems have shown the potential to improve the safety and efficacy of TiO_2_ NPs. However, clinical translation of nanomedicine-based therapies remains costly, complex, time-consuming, with additional challenges related to large-scale manufacturing, biocompatibility, and regulatory requirements (Hua et al. 2018), all of which can limit their use in clinical settings, despite demonstrating reasonably high therapeutic efficacy in preclinical settings.

Critically, no clinical trials have evaluated TiO_2_ NPs as a cancer therapeutic in humans, with all studies remaining at the preclinical stage. This is in contrast to other nanocarriers that have successfully been translated to the clinical setting. In 1995, the FDA approved liposomal doxorubicin (Doxil), an anthracycline with greater targeting capabilities and decreased toxicity. Between 2000–2010, further approval of polymer, liposomal, and inorganic particles followed, with the nab-paclitaxel module, Abraxane being the most well-known example. Immune-evading NPs came into development from 2011 onwards, and in 2017, CPX-351 (Vyxeos) became the first simultaneous dual-drug nanoplatform. Many of these drugs obtained their FDA approval as a result of their biocompatibility and high biodegradability (Venturini et al. 2025). Until cancer-specific human efficacy can be demonstrated, TiO_2_ and related nanoparticle systems remain considerably behind in clinical translation.

## Conclusion

TiO_2_ NPs have emerged as a promising platform for anticancer therapy due to their tunable physicochemical properties, photocatalytic properties, and drug delivery capacity. Their applicability in a wide range of drug targeting and release techniques such as surface functionalization, stimuli-triggered releases, and upcoming methods such as tumor targeting ligands and exosomes are also highly promising. Additionally, site-specific activation through photodynamic, photothermal, and sonodynamic cues provides a substantial advantage over traditional therapies such as chemotherapy and radiotherapy which struggle with tumor resistance, toxicity, and lack of tumor specificity. While TiO_2_ NPs demonstrate selective cytotoxicity towards cancer cells and tumor subtypes, the scope of clinical application remains limited due to several disadvantages. These include the need for administration of high dosages, potential accumulation of metal dopants and conjugates, and disruption of biological pathways which can lead to genotoxicity, chronic inflammation, induction of tumorigenicity in healthy cells, while also penetrating important biological barriers. Furthermore, much of the evidence surrounding safety is derived from preclinical data, with key factors such as toxicity over long periods and tumor selectivity yet to be tested extensively in vivo. Efforts must be made to balance both the therapeutic efficacy and safety when developing TiO_2_ NP platforms, as it holds great promise for effective therapeutic use in cancer.

## Data Availability

No datasets were generated or analysed during the current study.
